# Understanding the Role of GPCR Heteroreceptor Complexes in Modulating the Brain Networks in Health and Disease

**DOI:** 10.3389/fncel.2017.00037

**Published:** 2017-02-21

**Authors:** Dasiel O. Borroto-Escuela, Jens Carlsson, Patricia Ambrogini, Manuel Narváez, Karolina Wydra, Alexander O. Tarakanov, Xiang Li, Carmelo Millón, Luca Ferraro, Riccardo Cuppini, Sergio Tanganelli, Fang Liu, Malgorzata Filip, Zaida Diaz-Cabiale, Kjell Fuxe

**Affiliations:** ^1^Department of Neuroscience, Karolinska InstitutetStockholm, Sweden; ^2^Department of Biomolecular Science, Section of Physiology, University of UrbinoUrbino, Italy; ^3^Observatorio Cubano de Neurociencias, Grupo Bohío-EstudioYaguajay, Cuba; ^4^Department of Cell and Molecular Biology, Uppsala Biomedical Centre (BMC), Uppsala UniversityUppsala, Sweden; ^5^Facultad de Medicina, Instituto de Investigación Biomédica de Málaga, Universidad de MálagaMálaga, Spain; ^6^Laboratory of Drug Addiction Pharmacology, Department of Pharmacology, Institute of Pharmacology, Polish Academy of SciencesKraków, Poland; ^7^St. Petersburg Institute for Informatics and Automation, Russian Academy of SciencesSaint Petersburg, Russia; ^8^Department of Life Sciences and Biotechnology, University of FerraraFerrara, Italy; ^9^Department of Medical Sciences, University of FerraraFerrara, Italy; ^10^Campbell Research Institute, Centre for Addiction and Mental Health, University of TorontoToronto, ON, Canada

**Keywords:** G protein-coupled receptor, addiction, schizophrenia, depression, heteroreceptor complexes, oligomerization, serotonin receptor, dopamine receptor

## Abstract

The introduction of allosteric receptor–receptor interactions in G protein-coupled receptor (GPCR) heteroreceptor complexes of the central nervous system (CNS) gave a new dimension to brain integration and neuropsychopharmacology. The molecular basis of learning and memory was proposed to be based on the reorganization of the homo- and heteroreceptor complexes in the postjunctional membrane of synapses. Long-term memory may be created by the transformation of parts of the heteroreceptor complexes into unique transcription factors which can lead to the formation of specific adapter proteins. The observation of the GPCR heterodimer network (GPCR-HetNet) indicated that the allosteric receptor–receptor interactions dramatically increase GPCR diversity and biased recognition and signaling leading to enhanced specificity in signaling. Dysfunction of the GPCR heteroreceptor complexes can lead to brain disease. The findings of serotonin (5-HT) hetero and isoreceptor complexes in the brain over the last decade give new targets for drug development in major depression. Neuromodulation of neuronal networks in depression via 5-HT, galanin peptides and zinc involve a number of GPCR heteroreceptor complexes in the raphe-hippocampal system: GalR1-5-HT1A, GalR1-5-HT1A-GPR39, GalR1-GalR2, and putative GalR1-GalR2-5-HT1A heteroreceptor complexes. The 5-HT1A receptor protomer remains a receptor enhancing antidepressant actions through its participation in hetero- and homoreceptor complexes listed above in balance with each other. In depression, neuromodulation of neuronal networks in the raphe-hippocampal system and the cortical regions via 5-HT and fibroblast growth factor 2 involves either FGFR1-5-HT1A heteroreceptor complexes or the 5-HT isoreceptor complexes such as 5-HT1A-5-HT7 and 5-HT1A-5-HT2A. Neuromodulation of neuronal networks in cocaine use disorder via dopamine (DA) and adenosine signals involve A2AR-D2R and A2AR-D2R-Sigma1R heteroreceptor complexes in the dorsal and ventral striatum. The excitatory modulation by A2AR agonists of the ventral striato-pallidal GABA anti-reward system via targeting the A2AR-D2R and A2AR-D2R-Sigma1R heteroreceptor complex holds high promise as a new way to treat cocaine use disorders. Neuromodulation of neuronal networks in schizophrenia via DA, adenosine, glutamate, 5-HT and neurotensin peptides and oxytocin, involving A2AR-D2R, D2R-NMDAR, A2AR-D2R-mGluR5, D2R-5-HT2A and D2R-oxytocinR heteroreceptor complexes opens up a new world of D2R protomer targets in the listed heterocomplexes for treatment of positive, negative and cognitive symptoms of schizophrenia.

## Fundamentals of GPCR Heteroreceptor Complexes, Their Allosteric Communication and Their Function

The concept of allosteric receptor–receptor interactions in G protein-coupled receptor (GPCR) homo- and heteroreceptor complexes of the central nervous system (CNS) gave a new dimension to brain integration and neuropsychopharmacology (Fuxe et al., 1983, 1998, 2008c, 2010b, 2014d; Zoli et al., 1993; Liu et al., 2000; George et al., 2002; Guo et al., 2008; Han et al., 2009; Borroto-Escuela et al., 2012a). Allosteric receptor–receptor interactions made possible through receptor oligomerization lead to novel receptor dynamics during which the receptor protomers change their recognition, pharmacology, signaling and trafficking and novel allosteric binding sites can develop (Borroto-Escuela et al., 2011, 2012a; Fuxe et al., 2012a, 2014d; Fuxe and Borroto-Escuela, 2016). GPCR heteroreceptor complexes can also involve ion channel receptors, receptor tyrosine kinases (RTKs), sets of G protein interacting proteins, ion channels and/or transmitter transporters (Fuxe et al., 2007; Flajolet et al., 2008; Guo et al., 2008; Borroto-Escuela et al., 2012b, 2015c,d, 2016c; Fuxe and Borroto-Escuela, 2016; Di Liberto et al., 2017). The allosteric interactions in such dynamic higher order receptor complexes take place in an orchestrated spatio-temporal fashion and participate in learning and formation of molecular engrams for short and long term memory (Fuxe et al., 2014b; Borroto-Escuela et al., 2015a). In addition, the NMDA receptor complex is also now regarded as a multifunctional machine at the glutamatergic synapse involving extrasynaptic and synaptic D1R-NMDAR and D2R-NMDAR heteroreceptor complexes (Liu et al., 2000, 2006; Zhang et al., 2016). There is a need to improve our understanding of the molecular organization of the receptor oligomers, their allosteric communication and the features of the receptor interface (Borroto-Escuela et al., 2010c, 2014a; Tarakanov and Fuxe, 2010).

Recently the molecular basis of learning and memory was proposed on the reorganization of the homo- and heteroreceptor complexes in the postjunctional membrane of synapses leading to changes in the prejunctional receptor complexes to facilitate the pattern of transmitter release to be learned (Fuxe et al., 2014b; Borroto-Escuela et al., 2015a). Long-term memory may be created by the transformation of parts of the heteroreceptor complexes into unique transcription factors which can lead to the formation of specific adapter proteins which can consolidate the heteroreceptor complexes into long-lived complexes with conserved allosteric receptor–receptor interactions (Fuxe et al., 2014a,b; Borroto-Escuela et al., 2015a). Thus, the homo-heteroreceptor complexes are regarded as highly dynamic assemblies formed or disrupted by integrated synaptic and volume transmission signals. These events are necessary for learning, and can become transformed into a consolidated rigid state with conserved allosteric communication representing molecular engrams resulting in a major long term modulation of the neuronal networks. This molecular plasticity change, whether transient or long term, can then alter the patterns of outflow in the brain circuits and induce transient and long-term changes in behaviors and cognitive functions. In line with this hypothesis, blocking synaptic removal of GluA2-containing AMPA receptors prevents the natural forgetting of long-term memories (Migues et al., 2016).

Of special relevance for structural plasticity, for example in the dendritic tree and its spines, may be the recruitment of RTK to the heteroreceptor complexes formed, which may result, for example in synergistic increases in neurite densities and their protrusions in primary neuronal cultures (Flajolet et al., 2008; Borroto-Escuela et al., 2012b; Liebmann et al., 2016).

The concept of biased GPCR agonism meaning functional selectivity was developed by Kenakin (2007, 2008, 2011). The agonist stabilization of distinct active states in the receptor conformation was suggested to be the mechanism involved in producing activation of discrete signaling pathways of GPCRs. In 1983/1985, receptor–receptor interactions and their relevance for receptor diversity were presented based on studies on neuropeptide/dopamine (DA) interactions (Agnati et al., 1983a,b; Fuxe et al., 1983; Fuxe and Agnati, 1985). It is now clear through the demonstration of the GPCR heterodimer network (GPCR-HetNet; Borroto-Escuela et al., 2014a) that the allosteric receptor–receptor interactions dramatically increases GPCR diversity and biased recognition and signaling leading to enhanced specificity in signaling (Fuxe et al., 2014f; Borroto-Escuela et al., 2015b; Fuxe and Borroto-Escuela, 2016). The multiple origins of diversity and specificity in GPCRs were elegantly clarified in 2005 (Maudsley et al., 2005).

## Dysfunction of the GPCR Heteroreceptor Complexes Can Lead to Brain Disease

A dysfunction or a disruption of the D2R heteroreceptor complexes can be a molecular basis for a pathological change in brain circuits. For example, an increase in D2R function leads to alteration in the activity of glutamate prefrontal afferents (Fuxe et al., 2008c), followed by development of schizophrenic symptoms. Understanding these D2R heteroreceptor complexes and their dysfunction in schizophrenia can lead to new strategies for its treatment and for avoiding side-effects of antipsychotics known to mainly act as D2R antagonists (Seeman, 2010), including a way to optimize combined treatment or single use of heterobivalent drugs targeting the D2R heteroreceptor complexes in schizophrenia. This is inspired by the current findings of various types of D2R heteroreceptor complexes (Borroto-Escuela et al., 2014c; Ferraro et al., 2014; Fuxe et al., 2014e, 2015; Pinton et al., 2015b; de la Mora et al., 2016).

**Figure 1:**
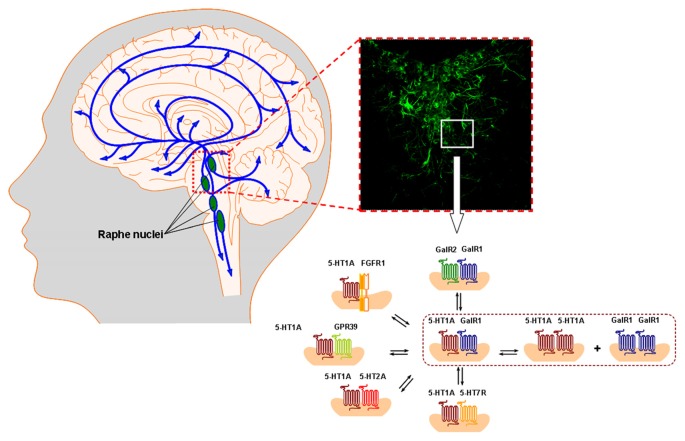
**The ascending and descending 5-HT pathways from the rostral and caudal raphe nuclei are illustrated in the left panel.** In the right panel the 5-HT immunoreactive nerve cell bodies and dendrites in the dorsal raphe are shown. The panorama of 5-HT1A heteroreceptor complexes including the 5-HT1A isoreceptor complexes demonstrated in the dorsal raphe and in the dorsal hippocampus are given in the lower right part shown as heterodimers and in possible balance with each other. They were described at the postjunctional level, likely in synaptic and extrasynaptic locations. The prejunctional existence of these 5-HT1A heterocomplexes at the 5-HT nerve terminal still remains to be studied. They play a major role in the 5-HT modulation of the neuronal networks in the raphe-hippocampal system together with the corresponding homoreceptor complexes and monomers. For the abbreviations see Supplementary Material.

**Figure 2:**
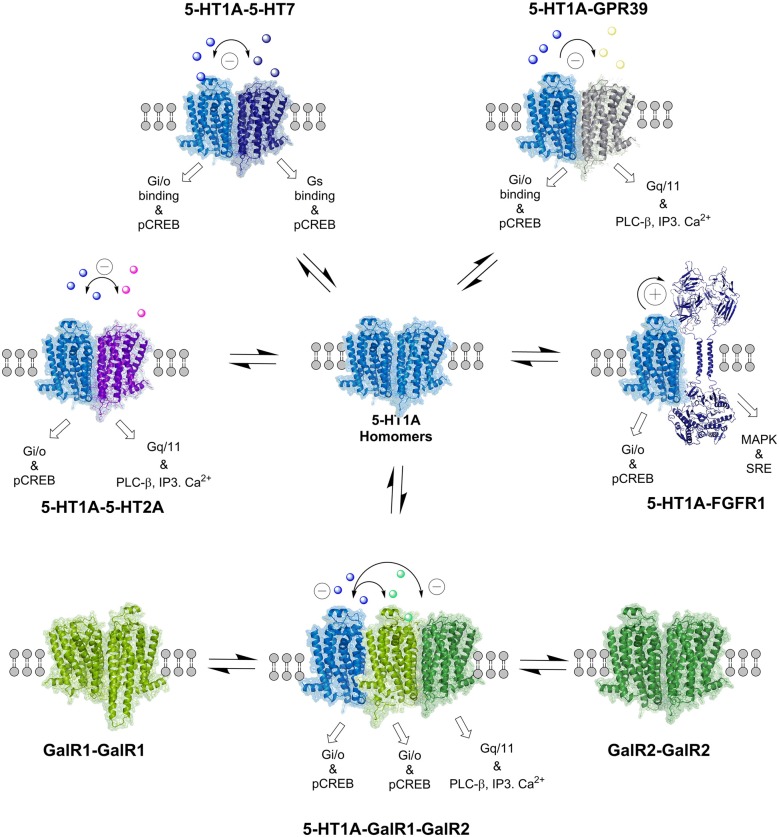
**The balance of the various serotonin 5-HT1A and galanin (GalR1 and GalR2) homo- and heteroreceptor complexes is indicated.** Also the 5-HT1A-FGFR1 heteroreceptor complexes are presented, which have a role in depression. The allosteric receptor–receptor interactions induced constitutively or by activation of one receptor protomer can influence the recognition, G protein coupling, signaling and trafficking of the other protomers in the homo and heteroreceptor complex as indicated. The nature of the allosteric receptor–receptor interactions found in the individual heteromers is indicated in the top part of the receptor complexes ([−] antagonistic allosteric modulation, [+] facilitatory allosteric modulation). The signaling of the receptor protomers reflects the signaling of the basal states. For the abbreviations see Supplementary Material.

With the discovery also of many 5-HT1A iso and heteroreceptor complexes, like the 5-HT1A-5-HT7 (Renner et al., 2012), the FGFR1-5-HT1A (Borroto-Escuela et al., 2012b, 2013a, 2015c,d, 2016c) and the putative trimer complex GalR1-GalR2-5-HT1A (Millón et al., 2014, 2016), an increased understanding of the molecular basis of major depression was obtained based on the 5-HT hypothesis of depression (Carlsson et al., 1968; Figures 1, 2). Postjunctional 5-HT1A receptors can strongly contribute to the mediation of the antidepressive effects of 5-HT (Artigas, 2015). It is of high interest that in these heteroreceptor complexes the receptor interacting proteins, like the scaffolding protein p11 (Svenningsson, 2014; Milosevic et al., 2017; Schintu et al., 2016), appear to play a substantial role (Fuxe and Borroto-Escuela, 2016). In human suicide victims reductions of p11 mRNA were established in hippocampus and amygdala and antidepressants enhance the expression of p11 in limbic regions of rodents (Svenningsson et al., 2013).

A significant role for the adaptor protein Disrupted in Schizophrenia in D2R heteroreceptor complexes has also been demonstrated (Su et al., 2014). This heterocomplex enhances D2R-mediated glycogen synthase kinase-3 signaling and reduces D2R internalization by agonists.

## GPCR Heteroreceptor Complexes as Targets for Drug Treatment in Brain Disease

The GPCR heteroreceptor complexes in the CNS have become exciting new targets for neurotherapeutics in Parkinson’s disease, schizophrenia, substance use disorder, anxiety and depression opening a new field in neuropsychopharmacology (Portoghese, 2001; Soriano et al., 2009; Le Naour et al., 2013; Fuxe et al., 2014e, 2015; Guidolin et al., 2015). Possible novel strategies for targeting heteroreceptor complexes in CNS disease are combined treatment with drugs targeting two receptor protomers. To enhance compliance of patients, fixed formulations of the two drugs can be developed which give optimal pharmacokinetics for a time window of long duration for the combined therapeutic actions. Dual acting drugs targeting two protomers in the receptor complex can also be developed as well as dual acting pro-drugs as a potential and novel multi-target approach to treat CNS disease (Borroto-Escuela et al., 2015e; Fuxe and Borroto-Escuela, 2015; Fuxe et al., 2015). Heterobivalent drugs are other options to selectively target heterodimers (Portoghese, 2001; Soriano et al., 2009; Gutiérrez-de-Terán et al., 2017) such as D2R antagonist and A2AR agonist pharmacophors targeting A2AR-D2R heterodimers in cocaine use disorder (Fuxe and Borroto-Escuela, 2016).

All these novel aspects on brain communication and integration in heteroreceptor complexes lead to increased understanding of the molecular basis of diseases in the CNS and of their treatments. There is increased demand to know and understand how the brain operates at the receptor level. The heteroreceptor field is novel since receptors are usually regarded to exist as monomers and this focused review will show that the receptor field has moved into homo and heterodimers and higher order homo and heteroreceptor complexes through use of novel methodologies (Borroto-Escuela et al., 2015b, 2016a). This research will therefore substantially advance the receptor field. It introduces a novel biological principle and neuropsychopharmacology which targets the heteroreceptor complexes. The homo-heteroreceptor complexes are also present *inter alia* in the peripheral nervous system, the endocrine, the cardiovascular and gastrointestinal systems. They represent new targets for drugs in molecular medicine.

## GPCR Heteroreceptor Complexes and Major Depression

**Figure 3:**
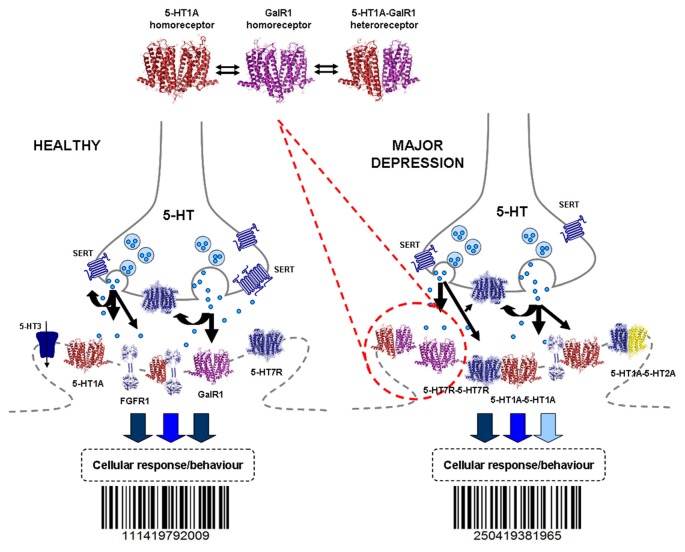
**The 5-HT1A hetero- and homoreceptor complexes shown as heterodimers are illustrated in the pre and postjunctional membranes of the 5-HT junctions.** This represents a general hypothesis not specifically related to the raphe and/or hippocampus. In the right part the reorganization of the panorama of the 5-HT1A heteroreceptor complexes in major depression are illustrated. Such reorganization can also take place in synapses and their peri and extrasynaptic regions including *inter alia* glutamate synapses involving also changes in the balance of the 5-HT1A heteroreceptor complexes with their corresponding homoreceptor complexes shown as hetero-and homodimers in the upper part. Also changes in 5-HT release may play a role in the 5-HT receptor reorganization. This leads to changes in the integrative 5-HT receptor signaling including the multiple 5-HT receptor families and the various heteroreceptor complexes formed from each of the 5-HT receptor subtypes. Thus, the barcodes of the 5-HT junctions and of *inter alia* glutamate and GABA synapses can be changed in depression. This induces changes in the operation of the neuronal networks e.g., the raphe-hippocampal 5-HT system contributing to changes in cellular and behavioral responses leading to major depression. For the abbreviations see Supplementary Material.

The 5-HT hypothesis of major depression was developed in the 1960s *inter alia* through the demonstration of the ascending 5-HT neurons from the midbrain innervating the entire tel- and diencephalon (Dahlstroem and Fuxe, 1964; Andén et al., 1966; Fuxe and Dahlström, 2009), biochemical studies on tryptophan and 5-HT (Coppen, 1967) and the 5-HT reuptake mechanism in these neurons as well as its blockade by imipramine (Fuxe and Ungerstedt, 1967; Carlsson et al., 1968). These observations led to the development of the 5-HT selective reuptake inhibitors (SSRI) for the treatment of depression. Over the decades a large number of 5-HT receptor subtypes were identified belonging to six families of G protein coupled 5-HT receptors, namely 5-HT1, 5-HT2, 5-HT4, 5-HT5, 5-HT6 and 5-HT7 receptors, while the 5-HT3 receptor is coupled to an ion channel (Barnes and Sharp, 1999). Even before their discoveries there were indications that classical antidepressants may block one particular type of 5-HT receptors (Fuxe et al., 1977; Ogren et al., 1979). Today we know that for antidepressant effect, 5-HT1A (postjunctional) or 5-HT4 receptors should be activated, while 5-HT2A, 5-HT3 and 5-HT7 should be blocked (Artigas, 2013). Thus, 5-HT receptor subtype selective antagonists and agonists or drugs acting at multiple 5-HT receptors and at serotonin transporter (SERT) can be used to enhance the antidepressant effects of SSRIs (Artigas, 2015). For the first time the hypothesis that the development of major depression can involve an imbalance of the receptor activity between different types of 5-HT isoreceptors was introduced in 1991 (Fuxe et al., 1991; Figure 3). Further findings of 5-HT hetero and isoreceptor complexes over the last decade give new targets for drug development in major depression. Below we discuss in detail the 5-HT hetero- and isoreceptor structures, functions and allosteric receptor–receptor interactions.

### GalR-5-HT1A Heteroreceptor Complexes

Early studies showed that in membrane preparations from the ventral limbic cortex Gal peptide (1–29) in the nanomolar range reduced the affinity of [^3^H]-5HT1A agonist binding sites (postjunctional sites) suggesting that Gal can reduce 5-HT1A recognition in this region (Fuxe et al., 1988). These interactions represent an integrative antagonistic mechanism in putative GalR-5-HT1A heteroreceptor complexes which may contribute to depression (Figures 1–3). The Gal receptor subtype was unknown. A role of raphe GalRs in depression and the use of general GalR antagonists as novel antidepressant drugs has been demonstrated (Bellido et al., 2002). An increased density of GalRs was found in the dorsal raphe of a genetic rat model of depression (Flinders sensitive line). It may contribute to the development of the depressive state though hyperpolarization of the 5-HT dorsal raphe nerve cells leading to reduced firing in the ascending 5-HT pathways to the forebrain. Again, the Gal receptor subtype is unknown.

### GalR1-5-HT1A Heteroreceptor Complexes

GalR1 homodimerization and internalization occurred in cellular models (Wirz et al., 2005), while GalR1-5-HT1A heteroreceptor complexes were found with FRET in HEK 293 cells (Borroto-Escuela et al., 2010b). The observed FRET ratios were unaltered by agonist treatments indicating that the heterocomplexes were constitutive. The results obtained with CRE-luciferase and SRE-luciferase reporter assays indicated possible antagonistic allosteric receptor–receptor interactions in this heteroreceptor complex. Thus, upon coactivation of the GalR1 and 5-HT1A protomers, both coupled to Gi/o, no additional inhibition of AC or stimulation of MAPK activity was observed in HEK293 cells. It is possible that in depression this antagonistic allosteric mechanism in the GalR1-5-HT1A heteroreceptor complexes is dysfunctional which may lead to disturbances in their operation in meso-limbic 5-HT neurotransmission.

### Dynamic GalR1-5-HT1A-GPR39 Complexes

GPR39 is a GPCR belonging to class A and represents a zinc binding receptor (Holst et al., 2007). Recent results demonstrated that zinc can disrupt the GalR1-5-HT1A heteroreceptor complex which may contribute to its antidepressant actions (Tena-Campos et al., 2015, 2016). In this process, GPR39 can form a dynamic heterotrimeric receptor complex with signaling diversity as demonstrated in cellular models (Tena-Campos et al., 2015). It should also be noted that GPR39 knockout mice show indications of depression (Młyniec et al., 2015) while zinc lowering produced a reduction of GPR39 expression (Młyniec et al., 2013). It appears that with high zinc levels, 5-HT1AR can no longer interact with GalR1 and the 5-HT1A-GPR39 heteroreceptor complex becomes the dominant form (Tena-Campos et al., 2015). The heterotrimer complex may represent an intermediate form dependent on zinc. This hypothesis offers one molecular mechanism for the antidepressant actions of zinc based on the ability of zinc binding receptor GPR39 to interact with the 5-HT1A-GalR1 heteroreceptor complex and changing the equilibrium towards GPR39-5-HT1A heteroreceptor complexes.

### GalR1-GalR2 Isoreceptor Complexes

Specific high affinity N-terminal Gal fragment (1–15) binding sites in contrast to the Gal (1–29) high affinity binding sites were early observed in the rat dorsal hippocampus, neocortex and striatum (Hedlund et al., 1992, 1994). These observations were made in spite of the fact that the three cloned Gal receptors (GalR1, GalR2 and GalR3), show a higher affinity for Gal than for Gal N-terminal fragments (Branchek et al., 1998). It was therefore proposed that the high affinity N-terminal Gal fragment binding sites developed in GalR1–GalR2 heteroreceptor complexes due to conformational changes in their GalR1 and/or GalR2 protomer recognition sites (Fuxe et al., 2012c).

In line with this hypothesis, GalR1–GalR2 heteroreceptor complexes were recently demonstrated in cellular models using BRET and in the midbrain raphe-dorsal hippocampal pathways of rodents using *in situ* PLA (Borroto-Escuela et al., 2014b). Evidence was obtained that Gal (1–15) had the ability to produce a disbalance of the signaling of the GalR1–GalR2 heterodimer with enhanced activation of the Gi/o mediated signaling via the GalR1 protomer while no significant effects were induced in the Gq/11 mediated signaling of the GalR2 protomer. Such a disbalance in the actions of Gal (1–15) on these isoreceptor complexes may contribute to depression-like actions since such effects are observed after treatment with GalR1 agonists (Fuxe et al., 2012c).

It is of substantial interest that a relevant role for the Gal N-terminal fragment (1–15) was found in anxiety- and depression-related behaviors (Millón et al., 2014). The strong depression-like and anxiogenic-like effects of the Gal fragment disappeared in the siRNA GalR1 and siRNA GalR2 receptor knockdown rats (Millón et al., 2014, 2016). The behavioral actions of Gal (1–15) were markedly reduced and related to the disappearance of the *in situ* PLA signals and, thus, to the disappearance of the GalR1-GalR2 isoreceptor complexes (Millón et al., 2014). Heterobivalent drugs with GalR1 and GalR2 antagonist pharmacophors may specifically target the GalR1 and GalR2 binding pockets of the GalR1-GalR2 heteroreceptor complex, disrupt their function and represent a new strategy for treatment of depression and anxiety.

### Putative GalR1-GalR2-5-HT1A Heteroreceptor Complexes

Gal (1–15) in contrast to Gal (1–29) markedly enhanced the antidepressant actions in the forced swimming test by the 5-HT1A receptor agonist 8-OH-DPAT (Millón et al., 2016) probably through alterations in the allosteric receptor–receptor interactions in the trimeric receptor complex. The effects involved Gal (1–15) induced effects in 5-HT1A receptors localized to the raphe-hippocampal 5-HT neuron system. It was possible to demonstrate the existence of GalR1-5-HT1A and GalR2-5-HT1A heteroreceptor complexes in the dorsal hippocampus and in the dorsal raphe. These results opened up the possibility that in fact the GalR1-GalR2 heteroreceptor complex can form a dynamic trimeric heterocomplex with the 5-HT1A receptor in these regions involving both 5-HT1A postjunctional and autoreceptors. If so, novel allosteric receptor–receptor interactions may be formed in these trimeric complexes that has led to enhanced 5-HT1A protomer signaling and enhanced anti-depressant effects in the forced swimming test (Millón et al., 2016). Gal (1–15) was found to increase the Kd and Bmax values and the mRNA levels of the postjunctional 5-HT1A receptors in the dorsal hippocampus but not of the 5-HT1A autoreceptors in the dorsal raphe. The 5-HT1A autoreceptor complexes appear to be differentially regulated with reduced mRNA levels in the dorsal raphe. Such events can lead to enhancement of 5-HT1A receptor signaling in the hippocampus and to enhanced firing of the ascending 5-HT neurons of the dorsal raphe. It is also possible that the 5-HT1A receptor agonist 8-OH-DPAT, via an allosteric receptor–receptor interactions, sets free Gq/11 signaling from the GalR2 protomer, known to have antidepressant actions (Lu et al., 2008), through allosteric inhibition of the GalR1 protomer signaling.

### Neuromodulation of Neuronal Networks in Depression via 5-HT, Galanin Peptides and Zinc Involving GPCR Heteroreceptor Complexes

It appears clear that different types of 5-HT1A heteroreceptor complexes containing also Gal R1, GalR2 protomers and/or GalR1-GalR2 isoreceptor dimers with or without GPR39 play a role in depression within the raphe-hippocampal system including the ascending 5-HT neurons to the tel- and diencephalon. The 5-HT1A receptor protomer remains a receptor involved in modulating antidepressant activity through its participation in the hetero and homoreceptor complexes listed above. The heterocomplexes are in balance with each other and exist especially in relation to the raphe-hippocampal neuronal system (Figures 1–3). The zinc binding receptor GPR39 may have an important role in this context that needs to be defined and further explored.

### 5-HT1A-FGFR1 Heteroreceptor Complexes

The demonstration of the FGFR1-5-HT1A heteroreceptor complexes in the hippocampus increased our understanding of how 5-HT1A receptors can enhance hippocampal plasticity (Borroto-Escuela et al., 2012b). Marked increases in neurite densities were *inter alia* found after agonist co-activation of these heterocomplexes and antidepressant actions developed upon combined icv treatment with FGF2 and a 5-HT1A agonist. It was associated with increased phosphorylation of FGFR1 and thus its activation, which can help counteract depression induced atrophy of the hippocampus (Borroto-Escuela et al., 2012b). A major neuromodulation appears to develop since the coupling of the 5-HT1A receptors to the GIRK channels appears to be reduced as well. This leads not only to increases in trophism but also to increased firing of hippocampal pyramidal nerve cells projecting directly or indirectly into the prefrontal cortex and the reward and anti-reward networks of the ventral striatum (Borroto-Escuela et al., 2015a, 2016c). Similar events appear to take place in the midbrain raphe with its rich presence of FGFR1-5-HT1A autoreceptor complexes of high relevance for neuroplasticity and depression (Borroto-Escuela et al., 2015c,d; Figures 1–3).

Due to FGFR1 protomer activation, the 5-HT1A autoreceptor function becomes diminished by its reduced coupling to GIRK channels and the firing in the dorsal raphe 5-HT neurons returns. Furthermore, the FGFR1 protomer activation can increase trophism leading to the outgrowth of new 5-HT nerve terminals including collaterals. Thus, the resulting enhancement of 5-HT communication probably produces antidepressant actions.

Similar events may also develop in the soma-dendritic regions of the noradrenaline (NA) and DA neurons of the locus coeruleus and ventral midbrain, respectively, in view of the local existence of putative FGFR1-alpha2A-adrenergic autoreceptor and FGFR1-D2 autoreceptor complexes. However, this possibility remains to be explored. It should be noticed that deep brain stimulation results in plastic changes of the central 5-HT neurons (Artigas, 2014; Veerakumar et al., 2014). We should consider that such increases in plasticity can involve activation of FGFR1-5-HT1A heteroreceptor complexes in the dorsal raphe and in the hippocampus as well as FGFR1-5-HT1B heteroreceptor complexes (Borroto-Escuela et al., 2012b, 2016c).

### Neuromodulation of Neuronal Networks in Depression via 5-HT and FGF2 Involving FGFR1-5-HT1A Heteroreceptor Complexes

An exceptional degree of neuromodulation can develop in ascending 5-HT pathways and in the hippocampus due to the existence of allosteric receptor–receptor interactions of FGFR1-5-HT1A heteroreceptor complexes in the midbrain raphe and in the pyramidal cells of the hippocampus. This modulation may correct both deficits in firing through antagonistic allosteric interactions by reducing the coupling of the 5-HT1A receptor to GIRK channels. With regard to trophism in the above regions, agonist-induced 5-HT1A activation appears to increase the trophic functions of the FGFR1 protomer (Borroto-Escuela et al., 2012b) reinstating trophic activity in prefrontal and reward networks of the forebrain. However, it is important to consider that in depression the allosteric receptor–receptor interactions in these heterocomplexes can be disturbed not only through dysfunction of the allosteric receptor–receptor interactions but also due to deficits in the formation of the FGFR1-5-HT1A heterocomplexes.

### 5-HT1A Isoreceptor Complexes

The first discovered 5-HT1A isoreceptor complex in cellular models was the 5-HT1A-5-HT7 complex in balance with 5-HT7-5-HT7 and 5-HT1A-5-HT1A homoreceptor complexes (Renner et al., 2012; Figures 2, 3) and the 5-HT1A-5-HT1B and 5-HT1A-5-HT1D complexes (Salim et al., 2002). Heterodimerization reduced the Gi/o mediated 5-HT1A signaling as well as the ability of 5-HT1A to open the GIRK channels, also demonstrated in the hippocampus. Furthermore, the heterodimerization was critically participating in the 5-HT induced internalization of the 5-HT1A protomer. It shows the impact of the 5-HT7 mediated allosteric receptor–receptor interactions on the 5-HT1A signaling and trafficking (Renner et al., 2012).

Very recently it was possible to demonstrate that the two major 5-HT receptor subtypes 5-HT1A and 5-HT2A (Celada et al., 2013) formed 5-HT1A-5-HT2A isoreceptor complexes in the rat cortical regions including the hippocampus (Borroto-Escuela et al., 2013c, 2016b). The 5-HT1A and 5-HT2A receptors are previously known to be co-expressed in cortical regions (Amargós-Bosch et al., 2004). The 5-HT isoreceptor complexes were demonstrated with *in situ* PLA and in cellular models also with BRET. The isoreceptor complexes were stress sensitive and markedly reduced upon exposure to a forced swim session in the hippocampus. An antagonistic allosteric receptor–receptor interaction appeared to be in operation since a 5-HT2A agonist upon activation of the 5-HT2A protomer markedly reduced the affinity of the 5-HT1A protomer. These results indicate that the 5-HT2AR having prodepressive properties can do so in part by directly inhibiting the recognition of the 5-HT1AR protomer, known to possess antidepressant actions upon activation (Celada et al., 2004). The early work in the 1970s showed that classical antidepressant drugs can block one type of 5-HT receptor (Fuxe et al., 1977; Ogren et al., 1979), likely the one identified as 5-HT2 (Peroutka and Snyder, 1979).

### Neuromodulation of Neuronal Networks in Depression via 5-HT Involving 5-HT Isoreceptor Complexes

Antagonistic allosteric receptor–receptor interactions take place in the 5-HT isoreceptor complexes discovered, at least with regard to the 5-HT1A protomer of the 5-HT1A-5-HT7 (Renner et al., 2012) and 5-HT1A-5-HT2A complexes (Borroto-Escuela et al., 2016b). Thus, in the modulation process there appears to exist a need in distinct states to produce an inhibition of certain functions of the 5-HT1A protomer to optimize the modulation of distinct networks.

The 5-HT1A receptor comes out as a hub receptor participating in a large number of iso and heteroreceptor complexes linked mainly to the raphe-hippocampal system of the brain (Salim et al., 2002; Borroto-Escuela et al., 2010d, 2014a; Gorinski et al., 2012; Renner et al., 2012; Tena-Campos et al., 2015). So far it is unknown which of the many 5-HT1A iso-heteroreceptor complexes show the major disturbance in major depression and should be the major target for novel antidepressant drugs.

## GPCR Heteroreceptor Complexes and Cocaine Use Disorder

**Figure 4:**
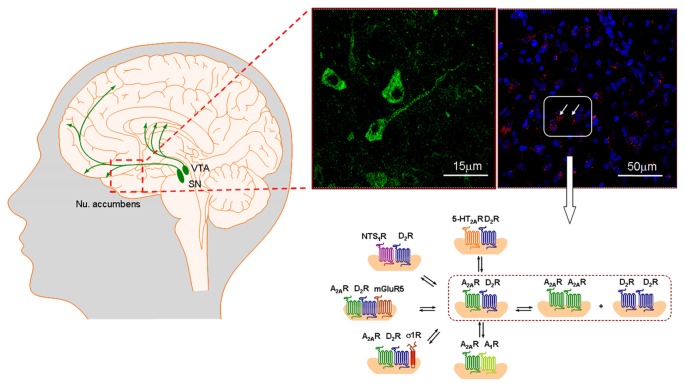
**To the far left the ascending nigro-striatal and meso-limbic-cortical dopamine (DA) neurons are presented.** The meso-limbic DA neurons play a major role in cocaine addiction *inter alia* through their dense innervations of the nucleus accumbens shell with their D2R positive nerve cells regulating the brain circuit from the nucleus accumbens shell to the prefrontal cortex. The first pathway is the ventral striato-pallidal GABA anti-reward system, rich *inter alia* in A2AR-D2R heteroreceptor complexes. In the far right part the A2AR-D2R heteroreceptor complexes are visualized as red clusters in the nucleus accumbens shell using the *in situ* proximity ligation assay (*in situ* PLA). To the left of this panel, sigma1R is seen as green immunofluorescence in nerve cell bodies in the nucleus accumbens shell which likely mainly represent GABAergic projection neurons. In this region a large panorama of D2R heteroreceptor complexes exists in the accumbens shell-ventral pallidal GABA anti-reward neurons modulating their activity (lower part). It represents previous work performed mainly in nuc accumbens (Fuxe et al., 2014e,f). They play as major role in modulating these neurons and the panorama is reorganized in cocaine addiction and schizophrenia contributing to the development of these diseases by increasing salience through *inter alia* removal of the brakes on the D2R protomer signaling found in distinct heterocomplexes. As examples from the nucleus accumbens are shown: A2AR-D2R, 5-HT2AR-D2R, NTS1-D2R complexes given as heterodimers and A2AR-D2R-mGluR5 and A2AR-D2R-sigma1R heterocomplexes given as heterotrimers (Cabello et al., 2009; Borroto-Escuela et al., 2010a,c,d, 2013b,c; Trifilieff et al., 2011). The A2A-D2 complexes are not located on the DA terminals and thus do not exist in a prejunctional position. Overall, the heteroreceptor complexes are mainly postjunctional on dendrites-soma but some are located also on glutamate terminals like A2AR-D2R and NTS1-D2R heteroreceptor complexes (Tanganelli et al., 2004, 2012).The scheme shown of heteroreceptor complexes in the figure does not itself give any indication of pre vs. post-junctional localization. For the abbreviations see Supplementary Material.

It is true that small reductions of long duration exist in striatal D2R availability and DA release of human cocaine addicts (Volkow et al., 2011). However, the relevance of D2R for cocaine use disorder was nevertheless shown in an article in which chronic cocaine self-administration increases behavioral responses mediated by D2Rs (Edwards et al., 2007). Furthermore, chronic cocaine self-administration evoked persistent more than 100% elevations of D2R binding sites in the high affinity type (Briand et al., 2008). Also D2R activation produced a strong relapse of cocaine seeking in rats (Self, 2010; Wydra et al., 2015b). The important role of D2R in cocaine use disorder was also demonstrated by the findings that chronic escalating dose “binge” of cocaine administration was associated with a D2R-stimulated G-protein activation and behavioral sensitization (Bailey et al., 2008). This was observed in spite of a decreased D2R density likely due to increased internalization (Bailey et al., 2008). D2R forms a large number D2R heteroreceptor complexes (Borroto-Escuela et al., 2014a; Fuxe et al., 2014e, 2015), some of which are of high relevance for understanding cocaine use disorder and its treatment (Figure 4).

### A2A-D2 Heteroreceptor Complexes

**Figure 5:**
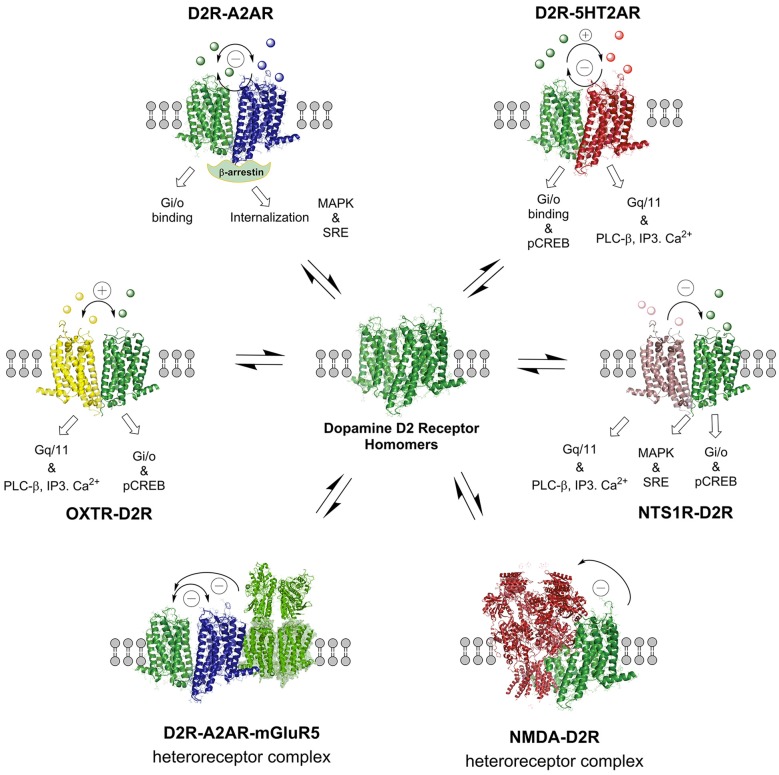
**The D2R heteroreceptor complexes illustrated in this figure are mainly found in the striatum.** The balance of the various D2R homo and heteroreceptor complexes are presented and their allosteric receptor–receptor interactions indicated. Also the NMDAR-D2R and OXTR-D2 heteroreceptor complexes are presented, the latter having a role in social salience. The basal signaling of some of the D2R heteroreceptor complexes is indicated. The nature of the allosteric receptor–receptor interactions found in the individual heteromers is indicated in the top part of the receptor complexes ([−] antagonistic allosteric modulation, [+] facilitatory allosteric modulation). For the abbreviations see Supplementary Material.

It is of high interest that A2AR-D2R heteroreceptor complexes with antagonistic allosteric receptor–receptor interactions were demonstrated in the ventral and dorsal striatum (Fuxe et al., 1998; Trifilieff et al., 2011; Borroto-Escuela et al., 2013c; Figures 4, 5). Such antagonistic allosteric A2AR-D2R interactions have been demonstrated at the neurochemical level (Frankowska et al., 2013; Wydra et al., 2015a) and in behavioral models, including cocaine reward and cocaine seeking in animals (Filip et al., 2006, 2012; Wydra et al., 2015b). Our latter findings support a role of A2ARs in modulating goal-maintained behaviors. They also indicate that increased accumbal GABA release via an antagonistic A2AR-D2R interaction can participate in mediating the inhibitory effects of the A2AR agonist on cocaine reward (Wydra et al., 2015a). Our results also indicate that A2AR activation and D2-like receptor blockade counteract cocaine and food relapse. It is proposed that A2AR- and D2R-mediated adenosine and DA signaling antagonistically interact in the ventral striato-pallidal GABA anti-reward neurons to regulate cocaine and food-seeking behavior (Wydra et al., 2015b).

Such an increase in antagonistic allosteric plasticity in the A2AR-D2R heteroreceptor complex can also help explain why the A2AR agonist CGS 21680 produced a dose-dependent blockade of the cocaine, quinpirole or cue induced reinstatement of cocaine seeking (Wydra et al., 2015a,b). Our findings strongly support the hypothesis that A2AR agonists targeting these A2AR-D2R heteroreceptor complexes can represent a novel treatment of cocaine use disorder.

### Higher Order A2AR-D2R Heteroreceptor Complexes: A2AR-D2R-sigma1R

Future work in the addiction research should *inter alia* focus on multiple A2AR-D2R heteroreceptor complexes including A2AR-D2R-sigma1R heterocomplexes (Navarro et al., 2010; Pinton et al., 2015a,b) and their balance with each other and the corresponding heterocomplexes. It is of high interest that cocaine induces a selective increase of sigma1 receptors in the ventral vs. the dorsal striatum (Romieu et al., 2002). Previous work demonstrated that the sigma1Rs are involved in mediating the pharmacological actions of cocaine and are potential targets for the treatment of substance abuse (Maurice and Su, 2009). The sigma1R is a chaperone protein located at the interface of the endoplasmic reticulum and the mitochondrium (Hayashi and Su, 2007). It regulates several functional proteins including D1R and D2R through oligomerization (Navarro et al., 2010, 2013) as well as voltage gated Kv1.2 potassium channels (Kourrich et al., 2013).

It is of interest that sigma1R-D2R heteroreceptor complexes can be formed while D3Rs and D4Rs cannot interact with sigma1R (Navarro et al., 2010). Multiple interfaces appear to exist between the sigma1R and D2R (Pinton et al., 2015a,b). These heteroreceptor complexes were found with *in situ* PLA in the ventral and dorsal striatum with highest densities in the dorsal striatum (Pinton et al., 2015a,b). Romieu et al. (2002) made the interesting observation that cocaine selectively increased the sigma1R in the ventral striatum. Instead Pinton et al. (2015b) and Borroto-Escuela et al. (2016d) found the preferential development of antagonistic A2AR-D2R interactions in the ventral striatum observed upon cocaine self-stimulation. Supporting our latter finding, cocaine (100 nM) enhances the D2R signaling in D2R-sigma1R heterocomplexes but not in D2R mono-homomers, likely representing allosteric modulations by cocaine induced by targeting the sigma1R protomer (Pinton et al., 2015a,b). Of particular relevance was the demonstration that the A2AR agonist CGS 21680 markedly reduced the ability of the D2R to inhibit the CREB signal in the A2AR-D2R-sigma1R heteroreceptor complex of the HEK293 cells in the presence of cocaine (100 nM; Pinton et al., 2015a,b; Borroto-Escuela et al., 2016d). Such trimeric heteroreceptor complexes may also exist in the nucleus accumbens shell in view of a considerable co-distribution of PLA positive A2AR-D2R and D2R-sigma1R heteroreceptor complexes in this region.

The marked increase of sigma1R in the plasma membrane induced by cocaine self-administration in the ventral striatum likely participated in the local significant return of the antagonistic allosteric A2AR-D2R interactions due to the increased formation of A2AR-D2R-sigma1R heterocomplexes. In this higher order receptor complex the A2AR-D2R interaction appeared to become stronger probably via strong cocaine-sigma1R interactions with restoration of significant and enhanced antagonistic A2AR-D2R interactions. The A2AR-D2R interactions were absent in the vehicle controls, since under these conditions the sigma1R may have a higher affinity for the D2R than for the A2AR-D2R heteroreceptor complexes (Borroto-Escuela et al., 2016d; Pintsuk et al., 2016b).

In contrast, in the dorsal striatum cocaine self-administration does not produce sigma1R recruitment to the plasma membrane (Romieu et al., 2002) and the antagonistic A2AR-D2R interactions disappear. The major change produced by cocaine may here be to elevate extracellular striatal DA levels which is postulated to increase the affinity for the formation of D2R homoreceptor complexes. As a result there is a reduction in the number of A2AR-D2R and A2AR-D2R-sigma1R heteroreceptor complexes (Borroto-Escuela et al., 2016d).

### Neuromodulation of Neuronal Networks in Cocaine Use Disorder via DA and Adenosine Signals Involving Multiple A2AR-D2R Heteroreceptor Complexes

The modulation of the ventral striato-pallidal GABA anti-reward neurons of the nucleus accumbens may have a critical role in addiction development. It involves the DA and adenosine volume transmission signals acting mainly on extrasynaptic D2R and A2AR homo and heteroreceptor complexes located on these anti-reward neurons. They are hub receptors and form a large number of hetero and isoreceptor complexes (Rocheville et al., 2000; Scarselli et al., 2001; Dunham et al., 2009; So et al., 2009; Borroto-Escuela et al., 2010e, 2014a; Przybyla and Watts, 2010; Verma et al., 2010; Espinoza et al., 2011; Van Craenenbroeck et al., 2011; Kern et al., 2012; Fuxe et al., 2014e, 2015; Figures 4, 5). The balance between the various D2R and A2AR hetero and homoreceptor complexes in the anti-reward system may vary from one neuron to the other and each neuron may have its own unique dynamic features in the panorama of receptor complexes available which will determine its neuromodulation. The ability of the individual neuron to form sigma1R may have a crucial role in its ability to mediate anti-cocaine action. The overall analysis so far strongly indicates that the excitatory modulation of this anti-reward system via targeting the A2AR-D2R and A2AR-D2R-sigma1R heteroreceptor complex in which the sigma1R can act as an adaptor protein holds high promise as a new way to treat cocaine use disorder (Figure 4). Further studies are requested to test how sigma1R agonists/antagonists may modulate the anti-cocaine actions of A2AR agonists in the modulation of the recognition and signaling of the A2AR-D2R heteroreceptor complexes.

## GPCR Heteroreceptor Complexes and Schizophrenia

Typical and atypical antipsychotics mainly act by targeting the DA D2 receptors in the brain (Seeman et al., 2002; Kapur, 2003; Ginovart and Kapur, 2010; Seeman, 2010). The critical D2Rs are linked to the meso-limbic DA neurons in the ventral tegmental area innervating densely, especially the nucleus accumbens core and shell (Dahlstroem and Fuxe, 1964; Fuxe, 1965; Andén et al., 1966). According to the DA hypothesis of schizophrenia, an increased activity of meso-limbic DA neurons contributes to an exaggerated release of DA from their nerve terminals mainly operating via extrasynaptic volume transmission (Fuxe et al., 2007, 2010a; Agnati et al., 2010). The resulting activation of the extrasynaptic D2Rs may excessively inhibit the ventral striato-pallidal GABA anti-reward neurons regulating a brain circuit to the prefrontal cortex as described above under “cocaine addiction” (Fuxe et al., 2008a,c, 2014e). Thus, it is important to state that a similar dysregulation of the ventral striato-pallidal GABA anti-reward neurons takes place in schizophrenia and in cocaine addiction.

Therefore, in schizophrenia with a silenced anti-reward system most stimuli obtain salience which may lead to symptoms like delusions in order to make sense out of the pathological interpretation of sensory inputs. The patients live in a world where all stimuli are of relevance (Kapur, 2003). A major action of the D2R antagonists should be to bring down salience without fully blocking it.

A breakthrough developed in the DA D2R field and its link to schizophrenia when different types of D2R heteroreceptor complexes were discovered in the forebrain (Fuxe et al., 1998; Hillion et al., 2002; Liu et al., 2006; Borroto-Escuela et al., 2013c, 2014c, 2016a; Figure 5). It opened up the possibility to pharmacologically reduce D2R function only in certain D2R heteroreceptor complexes that were disturbed in schizophrenia while others were not targeted by the novel antipsychotics to avoid side effects.

The D2R is a hub receptor which can interact with isoreceptors and other GPCRs (Borroto-Escuela et al., 2014a) but also with NMDAR (Liu et al., 2006), DA transporters and Disc1 (Su et al., 2014) which leads to diversity and bias (Fuxe et al., 2014f). Also receptor-protein interactions in higher order heteroreceptor complexes have become of importance in schizophrenia involving neuronal adhesion and scaffolding proteins formed from susceptibility genes (de Bartolomeis et al., 2013). The integrative process in higher order D2R heteroreceptor complexes have become increasingly complex and represent an important area for future research in schizophrenia.

Mainly the D2R-GPCR heteroreceptor complexes in the ventral and dorsal striatum will be discussed in relation to the ventral striato-pallidal GABA anti-reward neurons and their modulation of the brain circuit to the prefrontal cortex with impact on schizophrenia.

### Multiple A2AR-D2R Heteroreceptor Complexes

These heterocomplexes were already discussed in relation to cocaine addiction. In animal models of schizophrenia the A2AR agonist CGS 21680 behaves as an atypical antipsychotic (Rimondini et al., 1997). It can act as a negative allosteric modulator over the A2AR-D2R interface to reduce D2R protomer affinity in the high affinity state and Gi/o mediated D2R signaling in this heteroreceptor complex (Fuxe et al., 1998). A modest blockade of this D2R protomer will therefore more easily be obtained with reduced signs of depressive actions compared with D2R antagonists. The contribution of A2A agonist actions at the A2A monomer-homoreceptor complexes should also be considered in increasing activity in the striato-pallidal GABA anti-reward pathway (Fuxe et al., 2014c,d; Borroto-Escuela et al., 2015b,e, 2016d).

The amphetamine-induced sensitized state is a rat model of schizophrenia (Seeman et al., 2002). It therefore became of interest to study changes in the ventral and dorsal striatal allosteric A2AR-D2R receptor–receptor interactions in this model after an acute amphetamine challenge (Pintsuk et al., 2016a). As tested *ex vivo* with the A2AR agonist CGS 21680, there was a reinstatement of the antagonistic A2AR-D2R interaction in the ventral but not in the dorsal striatum after acute amphetamine treatment in the amphetamine sensitized state compared with the saline sensitized state. These actions of the A2AR agonist obtained in a rat model of schizophrenia can help explain its atypical antipsychotic profile (Pintsuk et al., 2016a). Instead the failure to produce antagonistic A2AR-D2R interactions in the dorsal striatum can assist to the development of the amphetamine-induced sensitized state shown as increases in locomotion.

It is of high interest that in this rat model of schizophrenia an increase in the D2R homodimerization was found in the dorsal striatum (Wang et al., 2010). Such an increase was also found in postmortem striatum from patients with schizophrenia. The absence of the antagonistic A2AR-D2R interactions in the dorsal striatum from rats in the amphetamine-induced sensitized state may therefore be due to the dominance of D2R homodimers over A2AR-D2R heteroreceptor complexes. It is suggested that the amphetamine-induced DA release in the dorsal striatum through the activation of the D2R orthosteric sites can enhance the affinity of the D2R protomers for each other leading to increased D2R homodimerization (Borroto-Escuela et al., 2015e, 2016d). A differential modulation of the A2AR-D2R heteroreceptor complexes and their balance with D2R and A2AR homoreceptor complexes likely develops in the ventral striatum of the amphetamine-induced sensitized state. It may be as seen after cocaine self-administration (Pintsuk et al., 2016b) that increases in sigma1R levels develop which can increase the formation A2AR-D2R-sigma1R with strong A2AR-D2R interactions.

The salience dysregulation in schizophrenia (Winton-Brown et al., 2014) appears linked to hyperactivity in the meso-limbic DA neurons (Grace, 2017) and to deficient brakes on D2R protomer signaling in its inhibition of the ventral-striato-pallidal anti-reward system involving *inter alia* multiple A2AR-D2R heteroreceptor complexes.

Also A2AR-D2R-mGluR5 heterocomplexes appear to exist in the ventral striato-pallidal GABA anti-reward pathway (Figure 5). In line with this view, it was indicated based on the codistribution of high densities of A2AR-D2R and A2AR-mGluR5 *in situ* PLA positive complexes in nucleus accumbens that A2AR-D2R-mGluR5 heterotrimeric complexes may exist in these anti-reward neurons (Borroto-Escuela et al., 2015b, 2016a). It was previously demonstrated in cellular models that A2AR-D2R-mGluR5 form trimeric heteroreceptor complexes using bimolecular fluorescence complementation and bioluminescence resonance energy transfer techniques (Cabello et al., 2009). They exist perisynaptically to glutamate synapses located on the striato-pallidal GABA neurons. The A2AR and mGluR5 protomers synergistically counteract the D2R recognition and signaling in these complexes (Ferré et al., 2002; Fuxe et al., 2003) and combined treatment with A2AR agonists and mGluR5 agonists/positive allosteric mGlu5 modulators should be a novel promising strategy for treatment of schizophrenia (Wieronska et al., 2016) with or without the use of D2R antagonists. Heterobivalent drugs built up of A2AR agonist and mGluR5 agonist/mGlu5 positive modulator pharmacophors should be interesting new drugs specifically targeting the A2AR-mGluR5 heteroreceptor complexes. Such compounds will also be of high interest for treatment of cocaine use disorder, since these heterocomplexes exist in the striato-pallidal GABA anti-reward system, highly relevant also for the treatment of cocaine use disorder (see above).

### Neuromodulation of Neuronal Networks in Schizophrenia via DA, Adenosine and Glutamate Signals Involving Multiple A2AR-D2R Heteroreceptor Complexes

As in cocaine use disorder, the ventral striato-pallidal GABA anti-reward system is of major importance also in schizophrenia. Major modulators of this anti-reward system are again DA and adenosine mainly operating via volume transmission and also glutamate operating mainly via synaptic but also via volume transmission. The balance of these signals and in the panorama of especially A2AR-D2R, A2AR-mGluR5, A2AR-D2R-mGluR5 and A2AR-D2R-sigma1R heteroreceptor complexes and their homoreceptor correlates will have a relevant impact on the activity of this anti-reward system and thus on the salience of the incoming sensory stimuli (Figure 4). It remains to be demonstrated which of these D2R heteroreceptor complexes are mainly disturbed in schizophrenia with a reduced brake on D2R protomer signaling leading to increases in schizophrenic symptoms. It should be considered that like in cocaine use disorder the synthesis and transport of the chaperone protein sigma1R to the plasma membrane can also play a relevant role in schizophrenia through its participation in a number D2R heteroreceptor complexes including A2AR-D2R-sigma1R. As discussed, there are indications that the sigma1R brings about an increase in the antagonistic A2AR-D2R interaction (Pinton et al., 2015a,b; Borroto-Escuela et al., 2016d).

### Multiple NMDA Heteroreceptor Complexes

There is an agreement that hypofunction of NMDA receptors play a significant role in the pathophysiology of schizophrenia contributing especially to the cognitive and negative symptoms of this disease (Balu, 2016). The anti-psychotic actions of mGluR5 activation discussed above also appear to involve facilitatory allosteric receptor–receptor interactions at mGluR5-NMDA heteroreceptor complexes within glutamate synapses (Wieronska et al., 2016).

Of high interest are also the findings of D2R-NMDA heteroreceptor complexes with the D2R protomer linked to the NR2B subunit (Liu et al., 2006). This group demonstrated that the allosteric receptor–receptor interaction produced a reduced ability of Ca^2+^/calmodulin dependent protein kinase II to bind to NR2B. As a consequence, a reduced phosphorylation of the NR2B subunit takes place followed by a decrease of NMDA receptor signaling. Of particular relevance for schizophrenia may be the existence of this complex in the glutamate synapses of the ventral striato-pallidal GABA anti-reward neurons.

### Neuromodulation of Neuronal Networks in Schizophrenia via DA and Glutamate Signals Involving Multiple NMDA Heteroreceptor Complexes

The neuromodulation of the ventral striatal-ventral pallidal-mediodorsal thalamic-prefrontal cortical circuit is again of special interest (Fuxe et al., 2008a,b,c) in view of its role in salience. Salience in schizophrenia becomes exaggerated due *inter alia* to the dysregulation of D2Rs in certain types of D2R heteroreceptor complexes. Also as a reduced function in certain types of NMDA heteroreceptor complexes located *inter alia* on the striato-pallidal anti-reward neurons. It is of substantial interest that overactivity of DA nerve terminals may release increased amounts of DA to diffuse into the glutamate synapses and its perisynaptic regions on the GABA anti-reward neurons to bring down NMDA receptor signaling and thus the glutamate drive on the anti-reward neurons. The D2R-NMDA heteroreceptor complexes may therefore be at the heart of the dysregulation of the anti-reward neurons in schizophrenia together with the perisynaptic A2AR-D2R-mGluR5 heteroreceptor complexes where inhibitory D2R signaling becomes preferred by the allosteric receptor–receptor interactions. In the glutamate synapse the D2R-NMDA heteroreceptor complex is proposed to be in dominance in the balance with the mGluR5-NMDA complex where the trimer mGluR5-NMDA-D2R can be an intermediate form.

It seems possible that certain anti-reward neurons may possess both D2R-NMDA and A2AR-D2R-mGluR5 heteroreceptor complexes in glutamate synapses and perisynaptic regions, respectively (Figure 5). They may be more efficiently silenced by over activated D2Rs in schizophrenia than many other anti-reward neurons and more strongly contribute to the schizophrenic symptoms.

### 5-HT2AR-D2R Heteroreceptor Complexes

These heteroreceptor complexes were demonstrated with the BRET technique in cellular models and then with *in situ* PLA in the dorsal and ventral striatum including nucleus accumbens core and shell (Borroto-Escuela et al., 2010d, 2013c, 2014c; Albizu et al., 2011). It is of high interest that the hallucinogenic but not standard 5-HT2A agonists produced an enhancement of D2R protomer signaling via the 5-HT2AR protomer in the 5-HT2AR-D2R heteroreceptor complex. It therefore seems possible that in schizophrenia a pathological allosteric enhancement of the D2R signaling takes place after 5-HT activation of the 5-HT2AR protomer, without use of hallucinogenic 5-HT2A agonists. Such a mechanism can be one factor that explains why the high potency of many atypical anti-psychotics like risperidone and clozapine to block 5-HT2ARs can be a valuable action (Meltzer et al., 1989, 2010). An inverse 5-HT2AR agonist pimavanserin exerts antipsychotic actions especially in Parkinson’s disease (Meltzer et al., 2010; McFarland et al., 2011). It may be that L-DOPA and D2R agonist treatments have produced alterations *inter alia* in the stoichiometry and in the allosteric receptor–receptor interactions of the D2R-5-HT2AR heteroreceptor complexes, which makes them more vulnerable to the blocking effects of this inverse 5-HT2AR agonist.

### Neuromodulation of Neuronal Networks in Schizophrenia via DA and 5-HT Signals Involving 5-HT2AR-D2R Heteroreceptor Complexes

Through extrasynaptic volume transmission, 5-HT may activate the 5-HT2AR protomer of the 5-HT2AR-D2R heteroreceptor complexes which in the nucleus accumbens core and shell may mainly be located on the ventral striato-pallidal GABA anti-reward neurons. It is unknown if these neurons are different from the anti-reward neurons containing the multiple A2AR-D2R heteroreceptor complexes or if these types of complexes overlap with each other as studied with the *in situ* PLA. It will be important to establish if each striato-pallidal GABA neuron has its own unique panorama of D2R heteroreceptor complexes located in distinct positions along the dentrites, soma and terminals. The question is which D2R heteroreceptor complexes show the highest vulnerability in schizophrenia.

### NTS1-D2 Heteroreceptor Complexes

Antagonistic NTS1-D2R interactions were early on discovered in the dorsal and ventral striatum (Agnati et al., 1983b; Von Euler and Fuxe, 1987) and later on shown to take place in NTS1-D2R heteroreceptor complexes in cellular models using BRET (Koschatzky et al., 2011; Borroto-Escuela et al., 2013b) and in the ventral and dorsal striatum using *in situ* PLA (Figure 4). Using CRE luciferase gene assay it was found that NTS1 activation produced a strong blockade of the D2R induced inhibition of the AC-PKA-CREB pathway (Borroto-Escuela et al., 2013b). The inhibitory modulation by NTS1 of D2R recognition and signaling appeared to have a major location in the cortico-accumbens glutamate terminals on the ventral striato-pallidal GABA anti-reward neurons leading to increased glutamate release and activation of the anti-reward neurons (Ferraro et al., 2008, 2012, 2014). Such antagonistic NTS1-D2R interactions at the soma-dendritic level of these neurons can also contribute to this activation of the anti-reward neurons and thus to the anti-psychotic like actions of NT peptides (Ferraro et al., 2014). Also facilitatory NTS1-NMDAR receptor–receptor interactions were observed in the nucleus accumbens that can contribute to the anti-psychotic actions based on the hypofunction of NMDAR in schizophrenia (Ferraro et al., 2012, 2014). In the ventral midbrain, however, they can contribute to propsychotic actions due to their activation of the meso-limbic DA neurons.

### Neuromodulation of Neuronal Networks in Schizophrenia via DA, NT Peptides, and Glutamate Signals Involving NTS1-D2R and Putative NMDA-NTS1 Heteroreceptor Complexes

Neurotensin peptides are formed *inter alia* in neurons of the striatum and communicate via volume transmission to activate especially NTS1 receptors to inhibit D2R signaling and enhance NMDA signaling in NTS1-D2R and putative NTS1-NMDA heteroreceptor complexes, respectively linked to the anti-reward neurons. Thus, this modulation by the NT peptides increases glutamate release and activity in the anti-reward neurons. In this way reduced salience develops and schizophrenic symptoms are diminished. It has not been determined how the NTS1-D2R *in situ* PLA positive clusters overlap with the A2AR-D2R and 5-HT2AR-D2R positive PLA clusters in the GABA anti-reward neurons enriched in the D2Rs. Nevertheless, the NTS1-D2R and NTS1-NMDAR complexes have a substantial location in the accumbens glutamate nerve terminals.

### D2-oxytocinR Heteroreceptor Complexes

In classical studies it was shown by Insel and Young (2001) and his group that striatal oxytocin receptors play a key role in inducing the pair bonding found in the monogamous prairie vole female and that the D2Rs play an important role in the social attachment process (Gingrich et al., 2000; Insel and Young, 2001; Young and Wang, 2004). Coactivation of D2R and oxytocin receptors appears essential for the social attachment (Fuxe et al., 2012b). Evidence was obtained that the molecular mechanism mediating the social salience was the formation of D2R-oxytocinR heteroreceptor complexes in the dorsal striatum and especially in the nucleus accumbens core as shown with *in situ* PLA (Romero-Fernandez et al., 2013; Figure 5). In these heterocomplexes oxytocin via allosteric receptor–receptor interactions markedly increased D2R recognition (increased affinity of the high affinity state and increased density of D2Rs) and increased D2R Gi/o coupling. These effects of oxytocin were all blocked by an oxytocin receptor antagonist L368,899 (Romero-Fernandez et al., 2013). Signaling in DA D2R-oxytocin receptor heterocomplexes were also studied (de la Mora et al., 2016). Oxytocin was found to enhance the D2R-like agonist quinpirole induced inhibition of the AC-PKA-pCREB signaling cascade and quinpirole enhanced the oxytocin induced increases in the activity of the PLCbeta-IP3-calcineurin pathway in cellular models. These results are highly relevant for the role of the oxytocin and DA interactions in social attachment and their anxiolytic effects in the amygdala of the rat (de la Mora et al., 2016).

### Neuromodulation of Neuronal Networks in Schizophrenia via DA and Oxytocin, Involving D2R-oxytocinR Heteroreceptor Complexes

The oxytocin pathways from the paraventricular nucleus and the DA pathways from the ventral midbrain project into the nucleus accumbens and the dorsal striatum where the oxytocin and DA nerve terminal networks co-distribute (Fuxe et al., 2010a, 2012b). They communicate mainly via volume transmission to act *inter alia* on the D2R-oxytocinR heteroreceptor complexes where they enhance the signaling of each other via allosteric receptor–receptor interactions. We postulate that the role of these integrative D2R-oxytocinR heterocomplexes is to markedly increase social salience by being located to a special component of the ventral striato-pallidal GABA neurons involved in regulating a brain circuit reaching into the prefrontal cortex. As a result of the activation of the D2R-oxytocinR heteroreceptor complex the information passing into the prefrontal cortex will produce social attachment and trust and the negative symptoms of schizophrenia may become markedly reduced. It was also proposed that oxytocin can induce antipsychotic actions (Caldwell et al., 2009) which appears to be true after being given to schizophrenic patients intranasally (Feifel et al., 2010; Feifel, 2012). Based on our hypothesis, the failure of D2R antagonists to reduce negative symptoms of schizophrenia is due to the blockade of D2R protomers in the D2R-oxytocinR heterocomplexes. For treatment of deficits in social interactions in schizophrenia, we propose as treatment strategy the use of heterobivalent drugs with oxytocin and D2R agonist pharmacophors that can specifically target these heteroreceptor complexes. It may also be possible to find D2R antagonists with low potency to block the D2R protomers of these heteroreceptor complexes. Another alternative is also the use of combined treatment with oxytocin and a D2R agonist which preferentially target the D2R protomer in the D2R-oxytocinR heterocomplex.

## Concluding Remarks

The neuromodulation is induced via the allosteric receptor–receptor interactions in isoreceptor and heteroreceptor complexes and changes in their balance with each other and homoreceptor complexes. Such a neuromodulation may play a major role in the regulation of brain circuits. This is true especially for neuronal networks of depression, addiction and schizophrenia. The most exciting development in this field is the plasticity of the synaptic and extrasynaptic heteroreceptor complexes at pre and postjunctional level (Fuxe and Borroto-Escuela, 2016) and of their balance as found e.g., after cocaine self-administration (Borroto-Escuela et al., 2016d). The transmitters working via synaptic and/or volume transmission (Fuxe et al., 2010a; Borroto-Escuela et al., 2015a) may have a significant role in determining this plasticity through changes in the formation and stability of the panorama of hetero and homoreceptor complexes in balance with each other. This plasticity may in fact be altered in CNS disease leading to altered neuromodulation and dysfunction of the brain circuits with deficits in learning and memory (Fuxe et al., 2014a,b; Borroto-Escuela et al., 2015a) and in emotions and motivation.

As new drugs for specifically targeting the heteroreceptor complexes heterobivalent compounds are being developed since several years with agonist and/or antagonist pharmacophors (Daniels et al., 2005; Waldhoer et al., 2005; Soriano et al., 2009; Huber et al., 2012; Hübner et al., 2016). Structure-guided development of heterodimer selective GPCR ligands is currently being performed targeting the NTS1-D2R heteromers (Hübner et al., 2016). The hope is that such types of drugs including also heterobivalent drugs that can target allosteric binding sites can become drugs for the future in the treatment of neurological and mental disorders.

## Author Contributions

We confirm and declare that all authors meet the criteria for authorship according to the ICMJE, including approval of the final manuscript and they take public responsibility for the work and have full confidence in the accuracy and integrity of the work of other group authors. All authors have substantially contributed to the conception or design of the work. Also they have participated in the acquisition, analysis and interpretation of data for the current review version. They have also helped revising it critically for important intellectual content; and final approval of the version to be published. In addition, they have contributed in this last version of the manuscript in writing assistance, technical editing, language editing and proofreading.

## Conflict of Interest Statement

The authors declare that the research was conducted in the absence of any commercial or financial relationships that could be construed as a potential conflict of interest. The handling Editor declared a shared affiliation, though no other collaboration, with one of the authors FL and states that the process nevertheless met the standards of a fair and objective review.
