# Supplementary material for: Understanding the Role of GPCR Heteroreceptor Complexes in Modulating the Brain Networks in Health and Disease
Source: Front Cell Neurosci. 2017 Feb 21;11:37. doi: 10.3389/fncel.2017.00037 (PMC5318393; doi:10.3389/fncel.2017.00037)
Supplement: Supplementary file 1 [file DataSheet_1.docx]

List of abbreviations

A – adenosine

A2A– adenosine A2A receptor

AC – adenylyl cyclase

AMPA – alpha –amino-3-hydfroxy-5-methyl-4-isoxazolepropionic acid

# BRET – Bioluminescence resonance energy transfer

CGS 21680 – 4-[2-[[6-Amino-9-(N-ethyl-β-D-ribofuranuronamidosyl)-9H-purin-2-yl]amino]ethyl]benzenepropanoic acid hydrochloride, an adenosine A_2_ receptor agonist

CNS – central nervous system

CRE – cAMP response element

CREB – cAMP response element-binding protein

CSF – cerebrospinal fluid

DA – dopamine

DA D2R/D2R – dopamine D2 receptor

DISC1 – Disrupted in Schizophrenia 1

FGFR – fibroblast growth factor receptor

FRET –  fluorescence resonance energy transfer

GABA – gamma amino-butyric acid

GalR – galanin receptor

G_i/o_  – G protein inhibitory/other

G_q_ – G protein which activates phospholipase C

GIRK– G protein-coupled inwardly-rectifying potassium channels

GluA– glutamate ionotropic receptor AMPA type subunit 1

GPCR – G protein coupled receptor

GPCR-HetNet– GPCR heteroreceptor network

GPR 39 – G protein coupled receptor 39

HEK 293 – human embryonic kidney cells 293

5-HT – 5 hydroxytryptamine

5-HT1A – 5-HT1A receptor is a subtype of the 5-HT2 receptor that belongs to the serotonin receptor family

5-HT2AR – 5-HT2A receptor is a subtype of the 5-HT2 receptor that belongs to the serotonin receptor family

IP3 – Inositol trisphosphate

Kv 1.2 – Potassium voltage-gated channel subfamily A member 2

L368,899 – a selective antagonist of the oxytocin receptor

MAPK – mitogen activated protein kinase

mGluR – metabotropic glutamate receptor

mRNA – a large family of RNA molecules that convey genetic information from DNA to the ribosome

NA – noradrenaline

NMDAR – N-methyl-D-aspartate receptor

NR2B – Glutamate [NMDA] receptor subunit epsilon-2, also known as N-methyl D-aspartate receptor subtype 2B

NTS1 – neurotensin receptor 1

NT– neurotensin

8-OH-DPAT– 8-HYDROXY-2-(DI-n-PROPYLAMINO)TETRALIN,

a 5-HT1A receptor agonist

OXTR – oxytocin receptor

pCREB – phosphorylated cAMP response element-binding protein

PKA – Protein kinase A, a class of cAMP-dependent enzymes

PLA – proximity ligation assay

PLC – phospholipase C

RTK – receptor tyrosine kinase

SERT – serotonin transporter

Sigma1R – sigma-1 receptor (σ1R), one of two sigma receptor subtypes, is a chaperone protein at the endoplasmic reticulum (ER)

siRNA – small interfering ribonucleic acid

SRE – splicing regulatory element

SSRI -– selective serotonin reuptake inhibitor
